# The Relationship between Axis Length Difference and Refractive Error in Unilateral Myopic Anisometropic Children Treated with Orthokeratology

**DOI:** 10.1155/2023/3110478

**Published:** 2023-09-04

**Authors:** Lu Sun, Zheng-Xuan Li, Shi-Peng Wang, Hong-Xin Song

**Affiliations:** ^1^Beijing Aier-Intech Eye Hospital, Beijing 100021, China; ^2^Beijing Tongren Eye Center, Beijing Institute of Ophthalmology, Beijing Tongren Hospital, Capital Medical University, Beijing Key Laboratory of Ophthalmology and Visual Sciences, National Engineering Research Center for Ophthalmology, Beijing 100730, China

## Abstract

**Purpose:**

To explore the correlation between the axial length (AL) difference (myopic and nonmyopic eye) and the refractive error in children with unilateral myopia anisometropia (UMA) and to elucidate its clinical application in the process of Ortho-K lenses review following nonstop wearing.

**Methods:**

This study retrospectively analyzed the data of 70 children with UMA (age, 8–15 years) whose myopic eyes were treated with Ortho-K lenses. The spherical equivalent refractive errors (SERE) of the myopic eye ranged from −0.75 D to −4.25 D, and astigmatism was no less than −1.50 D. In addition, SERE of nonmyopic eyes were no less than −0.50 D. AL, and the refractive data of both eyes were measured at baseline. A multivariate linear regression was used to analyze the relationship between the AL difference and refractive error, and paired *t*-test was used to analyze the changes in AL in both eyes.

**Results:**

Every 1 mm axial length change corresponds to −1.627 D (95% CI: −1.921 D, −1.333 D; *P* < 0.001) change in refractive error in children. The association between the AL change and the degree of myopia did not change with age (*P*=0.751). Among the 70 subjects, 51 (72.86%) had myopia in the right eye, and the 95% confidence interval (CI) for myopia occurring in the right eye was 62.4%–83.3%. The paired *t*-test showed that the average AL growth was significantly slower in myopic eyes treated with Ortho-K lenses than in nonmyopic eyes (*t* = 9.805, *P* < 0.001).

**Conclusion:**

Every 1 mm AL change would cause an average refractive error increase. Age did not influence the association between AL changes and the degree of myopia. The right eye is more likely to be affected in children with UMA. The Ortho-K lens treatment slowed down the growth of AL in the myopic eye in children with UMA.

## 1. Introduction

Myopia is a major cause of visual impairment, and a study in 2016 predicted that about half of the world's population will be myopic by 2050, with 10% being highly myopic [1]. Myopic anisometropia is defined as a between-eye difference in spherical equivalent refractive errors (SERE) of ≥1.00 D [2]. Unilateral myopic anisometropia (UMA) is a specific condition of myopic anisometropia in which an unequivocally myopic eye is paired with a “plano” (SERE = ±0.25 D) companion eye [3]. The anisometropia is believed to result from asymmetric axial length (AL) growth due to uneven progression of emmetropia in childhood, and the increase in AL is believed to be the main cause of anisometropia [2]. The Ojai Longitudinal Study [4] followed up 359 children aged 6–17 years and recorded their refractive status; 2.5% of them were found to have developed myopic anisometropia in this period. Another study reported the estimated prevalence of UMA as 0.7% in young adults (<19 years) [3].

Wearing glasses has been suggested as the best way to correct anisometropia [5]; however, many patients cannot tolerate the lens effect (a significant difference in the perception of image size) [6]. Compared with glasses, contact lenses can effectively reduce binocular aniseikonia [5] and are therefore widely used to correct anisometropia [7]. In recent years, the role of Ortho-K lenses in myopia control has been extensively studied, and they have been proven to be an effective, nonsurgical treatment for myopia correction [8, 9]. However, due to the physiological AL growth, it is difficult to know the true refractive error changes after wearing Ortho-K lenses. In this study, we theoretically resolved that issue. Therefore, this study is significant for children with myopia who are not willing to stop wearing the lenses and wonder about the effectiveness of the Ortho-K lens treatment.

In our study of children with UMA, the nonmyopic eye was used as the internal control of the fellow myopic eye (in the experiment of optical and drug therapy, this method is of great use for observation and comparative analysis). The aims of our study were to (1) describe the relationship between changes in AL and the refractive error by analyzing the data in children with UMA aged 8 to 15 years, (2) describe the distribution characteristics of children with UMA, and (3) observe the differences in the changes in AL in both eyes of children with UMA after wearing the Ortho-K lens. By fulfilling these aims, the current study endeavors to provide a feasible method for assessing the real myopia progression in children treated with Ortho-K lens.

## 2. Materials and Methods

### 2.1. Subjects

We retrospectively obtained the examination results from the medical records of 70 children with UMA (140 eyes) who met the inclusion criteria (Table 1) and whose myopic eyes were treated with Ortho-K lenses for more than 12 months in the myopia control outpatient clinic in Beijing Tongren Hospital between January 2017 and May 2020. This study adhered to the tenets of the Declaration of Helsinki and was approved by the Ethics Committee of Beijing Tongren Hospital.

### 2.2. Procedures

Before the children began to wear the Ortho-K lens, all of them underwent a baseline ocular examination, which included slit-lamp examination, uncorrected visual acuity, best-corrected visual acuity obtained with manifest refraction, fundus examination, corneal topography, corneal curvature, and AL measurement. Cycloplegic refraction was performed at the first visit. Cycloplegia was achieved with four drops of compound tropicamide (0.5% tropicamide and 0.5% neosynephrine; Santen, Japan) with 10-minute intervals. Ten minutes after the application of the fourth drop, autorefraction was performed three times (TOPCON, Japan, model: KR-8100), and the mean value was calculated. Noncontact partial coherence interferometer (IOL-Master; Carl Zeiss, Germany) was used to measure AL in both eyes at baseline and 3, 6, and 12 months after Ortho-K lens wear. At each visit, AL was measured three times and the mean value was calculated. The corneal parameters, including corneal curvature and astigmatism, were obtained using a corneal topographer (Medmont E300, Medmont International Pty Ltd., Australia) by the same professional technician, and the best image was extracted for analysis; this procedure was repeated at least three times. The AL difference between the two eyes and the SERE ratio at baseline were calculated.

### 2.3. Ortho-K Lens Fitting

The Ortho-K lenses were spherical 4-zone lenses (*α*ORTHO®-K, Boston, MA or Paragon CRT™, Paragon Vision Sciences, Gilbert, AZ). The nonmyopic eye did not undergo any treatment. When selecting the trial lens, the alignment curve radius was determined using the flat-K and corneal eccentricity, and then, a properly fitting lens was chosen based on the fluorescein pattern under the slit-lamp and corneal topography. For the myopic eye, the children were advised to wear Ortho-K lens every night for at least eight consecutive hours. Follow-up visits were scheduled for 1 day, 1 week, 1 month, 3 months, and every 3 months after Ortho-K lens wear.

### 2.4. Statistical Analysis

The gender and the affected side composition of the subjects were described in the form of number and percentage, and the 95% confidence intervals (CI) were calculated. The paired *t*-test was used to compare the distribution of basic characteristics and biological parameters between nonmyopic and myopic eyes. A scatter plot of the AL difference and refractive error was drawn, and Pearson correlation test was performed. For elucidating the relationship between the refractive error and AL difference, refractive error was used as the dependent variable and AL difference was used as the independent variable to construct a crude linear regression model and a multiple linear regression model adjusted for age, flat-K, and flat-E, and the residual distribution was tested. The multiple linear regression model was used to analyze the interaction between age and AL difference. The physiological AL growth in the nonmyopic eye during one-year follow-up was described, and 95% reference interval was calculated. Statistical analyses were performed using SPSS software (ver. 26.0; SPSS Inc., Chicago, IL) with the significance level established at two-sided *P* < 0.05.

## 3. Results

### 3.1. General Demographic Information

The ocular data of 70 children (mean age, 10.61 ± 0.24 years; age range, 8–15 years; 28 males (40%) and 42 females (60%)) with UMA were collected during 12 months (Table 2). Among them, 51 subjects (72.86%) had myopia in the right eye and 19 subjects (27.14%) in the left eye. The 95% CI for myopia occurring in the right eye was 62.4%–83.3%. The minimal and maximal SERE of myopic eyes were −4.25 D and −0.75 D, respectively, and the mean SERE value was −2.39 ± 1.05 D. Furthermore, steep-K and flat-E were significantly lower in myopic eyes than in nonmyopic eyes. The baseline AL and AL at one-year follow-up were significantly longer in myopic eyes than in nonmyopic eyes (*P* < 0.001).

### 3.2. Correlation between Baseline AL Difference and Refractive Error

#### 3.2.1. Univariate Linear Regression Analysis on AL Difference and Refractive Error at Baseline

**Figure 1:**
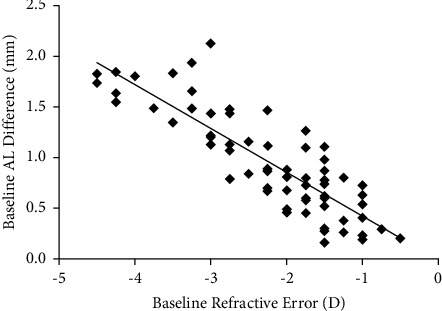
Scatter plot of baseline AL difference and refractive error.

The AL difference was correlated with the degree of myopia, and the degree of myopia increased with increases in the AL difference (Figure 1).

The model suggested a significant correlation between the AL difference and refractive error such that 67% of the variation in the refractive error could be explained by AL differences. For every 1 mm increase in the AL difference, the refractive error showed an average increase of −1.717 D (95% CI: −2.006, −1.428 D; *P* < 0.001; Table 3).

#### 3.2.2. Multiple Linear Regression Analysis on AL Difference and Refractive Error at Baseline

To further eliminate the influence of possible confounding factors and reveal the true association between baseline AL difference and refractive error, a multivariate linear regression model was established. The correlation analysis exhibited a strong correlation between baseline AL difference and refractive error, with a Pearson correlation coefficient of −0.853 (*P* < 0.001). It also revealed a correlation between the age and the refractive error, with a Pearson correlation coefficient of −0.410 (*P* < 0.001). The independent variables included in the regression model were baseline AL difference, age, flat-K, and flat-E in the myopic eye. The model explained 70.5% of the variation in the degree of myopia.

After adjusting for the age, flat-K, and flat-E in the myopic eye, the degree of myopia showed an average increase of −1.627 D (95% CI: −1.921, −1.333 D; *P* < 0.001) for every 1 mm increase in the baseline AL difference (Table 4).

#### 3.2.3. Correlation among Age, Between-Eye AL Difference, and Refractive Error

To further explore whether age affects the association between baseline AL difference and refractive error, we checked for an interaction term between the age and AL difference. The results showed that the association between the AL difference and refractive error did not change with age (*P* for interaction = 0.751; Table 5).

### 3.3. AL Growth in Nonmyopic Eyes

During the whole study period, all children maintained an uncorrected visual activity of 20/25 or better. The minimum and maximum physiological AL growths in nonmyopic eyes were 0.00 mm and 1.04 mm, respectively, and the median growth was 0.41 mm with an average increase of 0.44 ± 0.24 mm. The 95% reference interval of physiological increase of AL in nonmyopic eyes was 0.00–0.98 mm.

### 3.4. Changes in AL of the Two Eyes during One-Year Follow-Up

**Figure 2:**
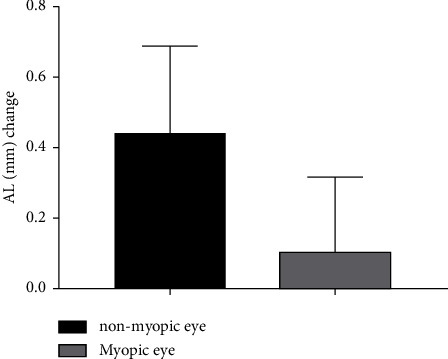
Changes in AL difference between the two eyes over a one-year period.

The average one-year AL growth was 0.443 ± 0.244 mm in nonmyopic eyes and 0.109 ± 0.208 mm in myopic eyes wearing Ortho-K lens (Figure 2). A paired *t*-test was used to compare the changes in AL between the two eyes over a one-year period. The result (*t* = 9.805; *P* < 0.001) indicated that the average increase in AL was significantly lower in myopic eyes than in nonmyopic eyes.

## 4. Discussion

Anisometropia is a unique refractive disorder, wherein two eyes of the same person develop asymmetrically, and consequently, the refractive power differs significantly between the two eyes. Therefore, anisometropia inherently supports the study of dose-dependent effects of an intervention on the two eyes of the same person by eliminating many intersubject variability aspects (such as age and environmental and genetic factors) [10]. It is essential to see the changes in biometry over time with nearly all variables controlled. The use of a contralateral eye study design in this study prevented the influence of potential confounding factors.

Previously reported data on the relationship between AL growth and myopia vary greatly. A study on the adult population by Badmus et al. [11] showed that a 1 mm increase in AL corresponds to a SERE increase of −0.77 D. Conversely, a study on adults aged over 40 years conducted by Olsen [12] showed that a 1 mm increase in AL corresponds to a SERE increase of −2.7 D. The differences in these results could be attributed to several factors, such as different geographic locations, older age, and different sample sizes.

A notable finding of our study is that, after adjustment for the age, flat-K, and flat-E in the myopic eye, the degree of myopia showed an average increase of −1.627 D for every 1 mm increase in the AL difference in children aged 8–15 years. In another study investigating 184 patients who underwent overnight Ortho-K treatment for 12 to 72 months and stopped wearing the lenses for 1 to 2 months, Chen et al. [13] found that 1 mm axial elongation was associated with approximately −1.60 D of myopia progression in children aged 6–14 years. However, they acknowledged some limitations of their study, in particular, the lack of a control group and the treatment span. As older children who demonstrated slower progression were also included in their study, the predictability of the regression model could have been compromised.

Because the nonphysiological growth of AL is instrumental in the progression of myopia, data on this nonphysiological growth are difficult to obtain in children after they start wearing Ortho-K lenses. Our current study included children with UMA, which allowed for the exploration of the real correlation between AL changes and the degree of myopia using the fellow nonmyopic eye as the control to eliminate the influence of physiological AL growth with age, and our observation span was one year, which can avoid confounding factors to some extent. Even though the sample composition of our two studies is different and the research methods have their own advantages and disadvantages, we have obtained consistent results from different research perspectives, which is strongly indicative of the reference value for the relationship between axial elongation and myopia progression.

A meta-analysis by Tsai et al. [14] summarized differences in the AL between the myopic eye and the healthy emmetropic eye in children with unilateral myopia after wearing the lens. The findings confirmed that Ortho-K lenses can efficiently reduce the intereye AL elongation difference in children with anisomyopia. The growth of AL has been confirmed to be lower in the myopic eye treated with an Ortho-K lens than in the healthy emmetropic eye [15], which corroborates our findings. Furthermore, long-term Ortho-K lens wear significantly reduces the between-eye AL difference in children with high anisometropia [16]. All the above studies have confirmed the efficacy of Ortho-K lenses in controlling anisometropic myopia.

To obtain the true refractive error after wearing Ortho-K lenses, it is necessary to stop wearing the lenses. However, to ensure continued myopia control effect, we do not normally recommend that children stop wearing them. Regular measurement of the AL allows for indirect monitoring of the increase in the degree of myopia, which may play a role in the efforts of myopia prevention and control. This study also provided an effective way to determine the effectiveness of the treatment using Ortho-K lenses, which is to compare the AL changes of this study as an uncontrolled group with the control group with lenses.

Studies have reported a strong correlation between the increase in AL and changes in refractive error [17], which is in agreement with the results of the current study, wherein we reported that refractive error increased with the increases in the AL difference between the myopic eye and the nonmyopic eye. In addition, our study reported that the AL increase in nonmyopic eyes in one year was 0–0.98 mm, with a median increase of 0.41 mm. Conversely, Chen et al. conducted a study encompassing a prolonged duration of 23.1 ± 8.3 months, wherein they reported the average annual AL growth in nonmyopic eyes as 0.39 ± 0.32 mm [18]. However, children with UMA may be a special group, and their nonmyopic eyes do not represent the range of normal value. In addition, different population compositions and different average age ranges will affect the results and return different research findings.

We noticed an interesting result in this study as follows: in the 70 children with UMA, the incidence in the right eye was significantly higher than that in the left eye. Although the sample size is limited, this finding is interesting.

In a previous study, AL was reported to increase logarithmically with age in children [19]. Therefore, age is an important factor in estimating SERE changes in myopia progression. To further determine whether the association between AL growth and myopia varies among children of different ages, we used the between-eye AL difference to correct the physiological growth of AL in the regression model. The result suggested that the association between AL difference and myopia was independent of age in children aged 8–15 years. This suggests that the change in refractive error corresponding to every 1 mm increase in AL should be the same in children aged 8–15 years.

In the research on the influence of Ortho-K lenses with different designs on peripheral refractive changes, Kang and Swarbrick [20] reported that Ortho-K lenses with different designs had a similar effect on myopia control as well. Therefore, our study did not consider the potential differences caused by differences in the designs of Ortho-K lenses.

The current study has a few limitations. First, it was a retrospective study with unavoidable selection bias and relatively small sample size. Therefore, the statistical power may not be sufficiently strong. Second, the study duration is relatively short. Although it is an internal control study, the results still need verification with more prospective studies. In the future, studies with larger sample sizes and longer follow-up periods are recommended.

## 5. Conclusions

In conclusion, every 1 mm AL difference in both eyes would cause an average refractive error increase of −1.627 D between the ages of 8 and 15 years. The age of the children had no effect on the association between the AL difference and the degree of myopia. The right eye is more likely to be affected in children with UMA. In addition, Ortho-K lens treatment is an optimal choice for the myopic anisometropic patients.

## Figures and Tables

**Table 1:** Inclusion criteria.

(1) The age was between 8 and 15 years at baseline
(2) Refractive error (spherical equivalent refraction) ranged from −4.25 D to −0.75 D in the myopic eye and between −0.5 D and +1.5 D in the fellow eye
(3) Best-corrected visual acuity was 20/20 or better in the myopic eye and the uncorrected visual acuity was 20/20 or better in the fellow eye (no correction was needed)
(4) Corneal power ranged from 40.00 D to 46.25 D and astigmatism was no less than −1.50 D
(5) There was no history of orthokeratology or use of other contact lenses or spectacles
(6) There were no serious eye diseases, such as congenital cataract, glaucoma, and strabismus and no active inflammatory ocular surface diseases
(7) There were no contraindications for overnight Ortho-K lens wear

Exclusion criteria were (1) SERE of the nonmyopic eye <−0.5 D or (2) uncorrected visual acuity of the nonmyopic eye ever dropped below 20/20 for any reason.

**Table 2:** Basic characteristics and biometric parameters of the participants.

Variables	Myopic eye	Nonmyopic eye	*P* value^*∗*^
Baseline AL (mm)	24.41 ± 0.11	23.44 ± 0.09	<0.001
CCT (*μ*m)	544.02 ± 4.33	546.37 ± 4.42	0.256
Flat-K	42.92 ± 0.16	42.90 ± 0.17	0.732
Steep-K	44.08 ± 0.17	44.20 ± 0.19	0.001
Flat-E	0.639 ± 0.012	0.662 ± 0.016	0.009
Steep-E	0.490 ± 0.027	1.378 ± 0.866	0.302
Astigmatism	−0.5 (−0.5, 0.0)	—	—
Pupil (mm)	6.14 ± 0.12	6.16 ± 0.11	0.682
1-y AL (mm)	24.51 ± 0.12	23.86 ± 0.10	<0.001

^*∗*^Paired *t*-test was used. AL = axial length; CCT = central corneal thickness.

**Table 3:** Univariate linear regression analysis on AL difference and refractive error at baseline.

Variable	Estimate	Standard error	95% CI	*P* value
Intercept	−0.770	0.154	(−1.078, −0.463)	<0.001
AL difference (mm)	−1.717	0.145	(−2.006, −1.428)	<0.001

The adjusted *R*^2^ was 0.670.

**Table 4:** Multiple linear regression analysis on AL difference and refractive error.

Variable	Estimate	Standard error	95% CI	*P* value
Intercept	6.094	2.504	(1.093, 11.096)	0.018
Age (years)	−0.078	0.037	(−0.152, −0.004)	0.040
Flat-K in myopic eye	−0.122	0.055	(−0.231, −0.013)	0.029
Flat-E in myopic eye	−1.418	0.795	(−3.005, 0.169)	0.079
Baseline AL difference (mm)	−1.627	0.147	(−1.921, −1.333)	<0.001

The adjusted *R*^2^ was 0.705.

**Table 5:** Multiple regression analysis on the interaction between age and baseline AL difference.

Variable	Estimate	Standard error	95% CI	*P* value
Intercept	5.832	2.652	(0.533, 11.131)	0.032
Age ∗ baseline AL difference	−0.024	0.074	(−0.171, 0.124)	0.751

The adjusted *R*^2^ was 0.754 (adjusted for age, AL difference, and flat-K and flat-E of the myopic eye).

## Data Availability

The datasets used and/or analyzed during the current study are available from the corresponding author on reasonable request.
